# Editorial: Text mining-based mental health research

**DOI:** 10.3389/frma.2024.1388691

**Published:** 2024-03-22

**Authors:** Tehmina Amjad, Tatsawan Timakum, Qing Xie, Min Song

**Affiliations:** ^1^Khoury College of Computer Science, Northeastern University, Silicon Valley Campus, San Jose, CA, United States; ^2^Department of Library and Information Science, Chiang Mai Rajabhat University, Chiang Mai, Thailand; ^3^School of Management, Shenzhen Polytechnic, Shenzhen, China; ^4^Department of Library and Information Science, Yonsei University, Seoul, Republic of Korea

**Keywords:** mental health, text mining, social media mining, topic modeling, sentiment analysis, deep learning, health informatics

In the field of data science, the power of computational and data analytics allows researchers to extract and interpret information from complex data in different domains. In medical research, it has enabled researchers to analyze large healthcare datasets, identifying meaningful knowledge using data mining and machine learning techniques. Currently, healthcare datasets are collected from laboratory data, scientific literature, and social media user-generated content. Social media has gained increasing popularity as a platform where individuals openly exchange experiences and information about various aspects of daily life, including healthcare details and discussions on mental health conditions. The discourse surrounding mental health has become very prominent in academia, reflecting progress in awareness of the challenges faced by people in maintaining psychological wellbeing. This issue delves into a multidimensional exploration of mental health research, leveraging innovative methodologies and technologies to strengthen our understanding and advance interventions in this critical area. This editorial provides a brief overview of the topics covered in this issue, one by one.

In the first article on the analysis of scientific literature datasets, Teijema et al. studied the use of active learning-based systematic review using deep learning models, as well as the impact of model switching strategies. The authors have performed a systematic literature review on a document collection based on query on psychological theories of depressive relapse. For performing a literature review, the selection of articles requires great human effort because relevant records are filtered from hundreds to tens of thousands of entries. Thus, this study utilizes the power of active learning-based methods to do this tedious job and shows the advantage of using active learning over manual screening for a large, labeled dataset. Then they developed a 17-layer conventional neural network and compared its performance with different combinations of classifiers. At the end, they performed model switching and analyzed that switching from a classical model like Naïve Bayes or logistic regression, to a deep neural network can improve the performance of the model. The dataset for this study is derived from a systematic review-based meta-analysis that examines the evidence supporting popular biological and psychological theories regarding the development, maintenance, and recurrence of depressive disorders. The study has investigated the data supporting prominent biological and psychological theories regarding the beginning, persistence, and recurrence of depressive diseases. The five primary psychological theories of major depressive disorder relapse and recurrence behavioral, psychodynamic, cognitive, diathesis-stress, and personality-based were examined in the analysis to examine the proposed causal relationship between the theories and depressive disorders. The study identified that model switching works well compared to default classification models. The authors recommend starting the review with a light model, such as logistic regression or Naïve Bayes, and transitioning to a stronger classification model based on a heuristic rule as required.

The article by Lezhnina explores the application of BERTopic to PubMed articles on depression, anxiety, and burnout in academia, unveiling evolving trends and areas warranting further investigations. Through comprehensive topic modeling, researcher studied the dynamic nature of mental health discourse within academic circles. This highlights emergent themes and highlights the need for targeted research efforts in this domain. The study reveals the strengths of BERTopic in dissecting the traces of mental health literature, shedding light on prevailing topics and trends from a dataset that comprises abstracts of 2,846 from PubMed articles ranging from 1975 to 2023. The researcher has employed a modular approach encompassing text embedding, dimensionality reduction, clustering, and weighing schemes to study and visualize 27 topics. Primarily, the study provides it readers a thorough explanation about benefits of BERTopic as a modular technique that permits flexible choices at every stage of document embedding, clustering, and topic development in studying mental health.

The study applied BERTopic models with different sets of parameters and compared them in terms of topic interpretability, the number of outliers, topic coherence and topic diversity. It was identified that research publications on burnout of medical residents and concerns of students related to pandemics have peaked in recent years. Publications on internet-related issues or psychometric research, however, still require greater focus.”

In the articles on the analysis of social media user-generated content datasets, Buddhitha and Inkpen investigated the use of artificial intelligence to identify individuals who are at risk for mental illness and suicidal thoughts based on social media posts. The authors have conducted experiments utilizing three datasets that represented users who self-reported diagnoses of single or multiple mental disorders and users diagnosed with varying levels of suicide risk and explored the association between mental diseases and suicidal ideation. They were able to identify features that are unique to the particular mental disorder or suicide ideation. Data were collected from various social media platforms to examine the effectiveness of using a shared representation to extract features between mental illness and suicide ideation detection tasks. The study also investigated the effect of comorbidity on suicide ideation and tested the generalizability of trained models. The results show that the predictive accuracy of suicide risk increases when using data from users diagnosed with multiple mental disorders compared to a single mental disorder. The study also highlights the influence of various mental diseases on suicide probability and finds a significant effect when using data from individuals with Post-Traumatic Stress Disorder (PTSD). The study used multi-task learning with soft and hard parameter sharing to identify users contemplating suicide and requiring immediate assistance. Cross-platform knowledge exchange and predefined auxiliary inputs increase the predictability of the proposed model. The article by Timakum et al. focuses on the Bipolar Reddit community, shedding light on the nuanced experiences and challenges faced by individuals living with bipolar disorder. Through the application of LDA topic modeling and sentiment analysis methods, the researchers have revealed the multifaceted dimensions of bipolar disorder and identified the role of social media discussion forums which serve not only as a tool for community building but also a platform for information dissemination. By analyzing over 1.4 million posts from the Bipolar subreddit community, researchers gained access to the thoughts, emotions, and experiences of the individuals, aiding in identifying the complexities of bipolar disorder. The study provides valuable insights into symptoms, mood swings, diagnosis, medication, therapy, and social support in this area. Along with the significant findings of the study, the authors have also identified the potential challenges in the area. Authors have identified the fact that social media discussions about bipolar disorder involve various participants, such as diagnosed individuals, self-identifying individuals, and family members. This diversity poses a risk of misinterpretation when analyzing sentiments and topics related to bipolar disorder.

In conclusion, to effectively traverse the challenging field of mental health research, interdisciplinary collaboration and knowledge exchange are essential. The convergence of text mining, social media analysis, and machine learning approaches presents unprecedented opportunities for comprehensive understanding and action in mental healthcare. By leveraging technology's transformative potential, we can address urgent mental health issues and empower individuals on their journey toward wellbeing by integrating insights from multiple study disciplines.

To sum up, the research presented in this issue highlights the revolutionary potential of multidisciplinary investigation and technological advancement in the progression of mental health studies. The approaches from machine learning and active learning techniques to text mining and social media analysis offer diverse perspectives and methods for clarifying the nuances of mental health discourse and intervention. As we embark on this collective endeavor, let us stay dedicated to utilizing the entire range of knowledge and experience to promote a more diverse, caring, and resilient community. The combination of text mining, social media analysis, and machine learning can yield valuable insights that can guide evidence-based therapies, raise public awareness of mental health issues, and cultivate a culture of understanding and empathy for all.

## Author contributions

TA: Writing – original draft, Writing – review & editing. TT: Writing – original draft, Writing – review & editing. QX: Writing – review & editing. MS: Writing – review & editing.

